# The Emerging Role of MR Urography in Imaging Megaureters in Children

**DOI:** 10.3389/fped.2022.839128

**Published:** 2022-03-23

**Authors:** Dominik Świȩtoń, Małgorzata Grzywińska, Piotr Czarniak, Andrzej Gołȩbiewski, Agata Durawa, Jacek Teodorczyk, Mariusz Kaszubowski, Maciej Piskunowicz

**Affiliations:** ^1^Second Department of Radiology, Medical University of Gdańsk, Gdańsk, Poland; ^2^Department of Human Physiology, Medical University of Gdańsk, Gdańsk, Poland; ^3^Department of Paediatrics, Nephrology and Hypertension, Medical University of Gdańsk, Gdańsk, Poland; ^4^Department of Surgery and Urology for Children and Adolescents, Medical University of Gdańsk, Gdańsk, Poland; ^5^Department of Nuclear Medicine, Medical University of Gdańsk, Gdańsk, Poland; ^6^Faculty of Management and Economics, Department of Statistics and Econometrics, Gdańsk University of Technology, Gdańsk, Poland; ^7^First Department of Radiology, Medical University of Gdańsk, Gdańsk, Poland

**Keywords:** MR urography, megaureter, hydronephrosis, children, CAKUT

## Abstract

**Introduction:**

Megaureter, described as ureter dilatation more than 7 mm in diameter, commonly associated with other anomalies, is still a diagnostic and therapeutic challenge. Magnetic resonance urography (MRU) appears as a promising method in urinary tract imaging, providing both anatomical and functional information. There are several postprocessing tools to assess renal function (including differential renal function) and severity of ureteral obstruction based on MRU. Still, the place of this method in the diagnostic algorithm of ureteropelvicalyceal dilatation with megaureter remains underestimated. Analysis of imaging findings in a group of children diagnosed with megaureter was done.

**Material and Methods:**

A retrospective analysis of magnetic resonance urography (MRU) was performed in 142 consecutive patients examined from January 2013 to September 2019. Twenty-five patients meeting the criteria of megaureter (dilatation more than 7 mm) in MRU were included in the further analysis. The MRU, ultrasound (US), and scintigraphy results were compared and analyzed together and compared with clinical data.

**Results:**

The sensitivity and specificity of US was comparable to the MRU in the assessment of upper urinary tract morphology (*p* > 0.05). In five out of 25 children, megaureter was found in each kidney; in a single case, both poles of a duplex kidney were affected. In the diagnosis of ureter ectopia, the MRU was superior to the US for which sensitivity did not exceed 16%. The US showed limited value in the diagnostics of segmental ureter dysplasia as a cause of primary megaureter when compared with MRU. Four cases were visualized in MRU studies, whereas the US examination was negative (all confirmed during surgery). There was a moderate correlation between relative renal function between fMRU and scintigraphy (*t* = 0.721, *p* = 0.477) and in the severity of obstruction assessment between both methods (*r* = 0.441, *p* < 0.05). However, in 10 kidneys with megaureter, the results in scintigraphy were inconclusive due to the signal from the megaureter imposing on the renal field.

**Conclusions:**

MRU seems to be a preferred method in the diagnostic algorithm for megaureter, providing both anatomical and functional information. MRU is superior to US and scintigraphy in diagnosing urinary tract anomalies with megaureter.

## Introduction

Megaureter is a descriptive term used for a ureter that is widened by more than 7 mm. This term covers a wide spectrum of pathologies, often associated with other anomalies, such as duplex kidney, ectopic ureteral insertion, and vesico-ureteral reflux as well as an isolated finding (1). The basic classification distinguishes between the refluxing and non-refluxing origin of the ureter dilatation, which implies further assessment (1–3). Most patients are diagnosed prenatally, but the direct cause of dilated ureter is rarely defined at this stage. Ultrasound (US) usually remains an initial diagnostic tool; however, a further diagnostic approach is often required. According to urological guidelines, voiding cystourethrography (VCUG) and dynamic renal scintigraphy with ^99m^Tc-mercaptoacetyltriglycine (MAG3) are the recommended tools for diagnostic management of patients with megaureter (2, 3). However, these imaging techniques allow the visualization of the exact anomaly of the ureterovesical junction (UVJ) or the severity of obstruction only in a limited percentage of cases. Due to the rapid technological advancements in radiology, including new options of US or magnetic resonance urography (MRU), diagnostic algorithms have evolved dramatically. New diagnostic modalities are particularly favorable because both examine the VCUG, and renal scintigraphy exposes patients to ionizing radiation (4). Therefore, this study presents the potential benefits of the implementation of advanced MRI techniques in the diagnostics of obstructive uropathy due to UVJ obstruction with the focus on children primarily diagnosed with megaureter.

**Figure 1:**
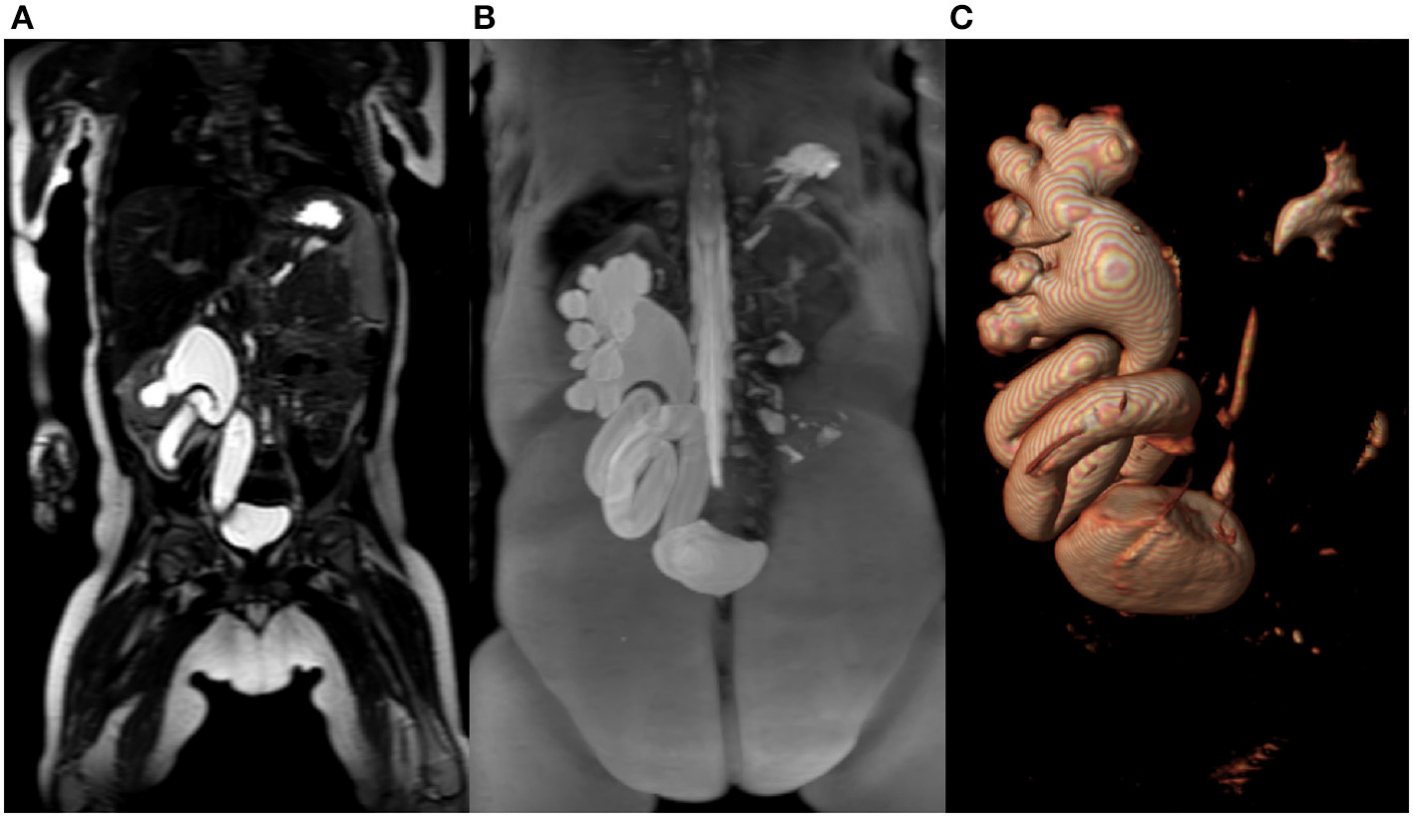
**(A)** T2-weighted- image in coronal plane of right kidney with megaureter. **(B)** Maximum intensity projection (MIP) reconstruction image of the right kidney collecting system with megaureter. **(C)** The same patient with reconstruction in 3D-volume rendering technique.

MRU is based on two techniques: (1) non-contrast heavy T2 weighted imaging, termed hydrography, and (2) T1 imaging following contrast administration: functional MRU (fMRU) (5, 6). The first technique allows the visualization of the dilatated renal collecting system anatomy with possible 3-D reconstructions, which are especially useful for surgeons (Figures 1A–C). This method is non-invasive, can be performed regardless of renal function, and does not pose any threat of allergic complications as no contrast agents are required. However, the functional evaluation is needed if conservative treatment and observation are considered, which is often the case, as spontaneous remission can be expected in up to 85% of patients (7). The information about renal function and severity of obstruction can be obtained by the means of the renal scintigraphy, intravenous urography, pyelography, or fMRU based on dynamic sequences after intravenous contrast administration. The first three techniques involve radiation exposure and, according to the ALARA principle, should be replaced by radiation-free imaging whenever possible, especially for pediatric patients. The fMRU is the most advanced technique providing information on renal anatomy and function, independently of the degree of collecting system dilatation. There are several tools for postprocessing fMRU images, allowing the assessment of renal function (including split renal function) and severity of obstruction; however, their usability is still being discussed (6, 8–10).

MRU provides additional information on the complications of obstructive uropathy, such as ectopic ureteral insertion, renal dysplasia, and parenchymal scars (11, 12). Despite the benefits of fMRU in the imaging of the ureteropelvicalyceal dilatation, its role in the diagnostic algorithm for children with megaureters is still unclear.

The purpose of the study is the analysis of the MRU findings in children with megaureters in terms of added value for diagnostic algorithm.

## Materials and Methods

### Study Group

The study was approved by the Independent Ethics Committee (NKBBN/22/2020).

A retrospective analysis of 142 MRU examinations for the period from January 2013 to September 2019 was done. A group of 25 patients with megaureter was selected for additional analysis. The presence of the megaureter, defined as ureter dilatation more than 7 mm, was the main inclusion criterion. The presence of the megaureter in MRU was the main inclusion criterion. All patients underwent MRU with hydrographic sequences and intravenous contrast administration Gadovist (Bayer AG, Germany), and most also had Tc-99m MAG3 scintigraphy. Qualification for surgery, clinical doubts, or discrepancies in the previous studies were the main indications for these studies.

### MRI

The examinations were performed with a Philips Achieva 3T TX magnetic resonance scanner (Philips Healthcare; Best, The Netherlands) incorporating a 16-channel dedicated abdominal coil. MR protocol included the following sequences: T2W_TSE_Tra_HR; STIR_Tra_FB; VISTA_COR_Sense; mDixon_Tra;DWI_5b_Tra_navi; sMRCP_3D_HR_COR; e-THRIVE_COR_FB; mDixon_Tra C+.

The dynamic sequence of choice was 3-D thrive (Enhanced T1 High-Resolution Isotropic Volume Excitation). This sequence was optimized (regarding the field of view (FOV) and number of slices) for each patient to improve spatial and temporal resolution.

The time of examination was up to 40 min (depending on patient breathing frequency with breathing triggering option), including a dynamic sequence, which took 15 min on average. All children received intravenous infusion of at least 10 ml of normal saline per kg of body weight 1 h before the study.

The diuretic (Furosemidum Polpharma, Polpharma SA) at the dose of 0.25–0.5 mg/kg up to 15 mg was administered 15 min before contrast injection.

Children younger than 5 years old were examined under general anesthesia.

### Postprocessing

The results of the MRI dynamic sequence were analyzed with the available postprocessing software: chop-fMRU dedicated to MR urography analysis (10).

The analysis in the chop-fMRU software was divided into a few stages: (1) Segmentation of the aorta and kidneys in the arterial phase after contrast material administration. (2) The analysis of the contrast-enhanced dynamic sequence with generation of renal parenchymal signal intensity time curves throughout the nephrogenic and excretory phases. (3) The analysis of quantitative parameters related to contrast material transit from aorta to renal collecting system. The main parameters reported include calyceal transit time (CTT), renal transit time (RTT), automatic calculation of the differential renal function (DRF) based on volume-enhanced parenchyma (vDRF), the Patlak numbers, a functional parameter per unit of tissue (pDRF). (4) The analysis of vpDRF (volume Patlak differential renal function) as the most adequate for renal function estimation, including information about the efficiency calculated per volume of the kidney. If there was a duplex collecting system, vpDRF was additionally calculated for each renal moiety. The results were compared with MAG3 renal nuclear scans reports if available. The vpDRF was compared with scintigraphy split renal function (SRF). Additionally, RTT was compared with time to t1/2 time in MAG3 nuclear scans. RTT is the time of contrast transit until it appears in the ureter at the level of the lower pole of the kidney. The group was divided into three subgroups to determine the severity of uropathy obstruction with RTT < 4 min considered normal, 4 < RTT < 8 min considered moderate obstruction, and RTT > 8 min indicating significant obstruction (13).

### Ultrasound Imaging

The US reports were analyzed 3 months prior to MRU exams. The exams were conducted by two physicians experienced in renal US (P.C.–pediatric nephrologist with 25 years of experience, D.S.–pediatric radiologist with 15 years of experience). We used two US units: GE Voluson S8 (GE Medical Systems, Milwaukee, WI, USA) and Philips Epiq 5 (Philips Ultrasound, Bothell, WA, USA). The exams were performed with linear, high-frequency probes 12–18 Mhz and convex 1–6 Mhz.

Findings such as duplex kidney, ureterocele, ureteral adynamic segment (dysplastic segment), or ectopic ureteral insertion were reported.

In both methods, MRU and US reports included size of the kidneys, cortico-medullar differentiation, classification into single or duplex kidney. The renal parenchyma was analyzed for scars or focal dysplasia presence. In MRU, the presence of the focal abnormal parenchymal signal with the presence of cysts was defined as dysplastic lesions. Focal scars were defined as thinning of the parenchyma with abnormal enhancement and/or abnormal decreased signal in T2WI and/or disturbed focal enhancement after contrast administration in T1WI. In US, the scars were defined as focal thinning of the renal parenchyma with or without changes in the echogenicity, whereas dysplasia was defined when the renal parenchyma structure was focally disturbed with cystic lesions.

The special attention was paid to megaureter morphology such as the presence of intramural stenosis, adynamic segment of the properly inserted ureter, ureterocelic orifice, or ectopic ureteral insertion.

### Renal Scintigraphy

Images were acquired with a dual-headed gamma camera system (Symbia T6, Siemens, Erlangen, Germany) with a low-energy, high-resolution collimator with an energy window centered at 140 keV for ^99m^Tc imaging. Infants and children received hydration (i.v. or p.o.) of 10 ml/kg of body weight starting 1 h before the study.

The dose of ^99m^Tc-MAG3 radiotracer, examination protocol, and data acquisition were done in accordance with the recommendations of the European Association of Nuclear Medicine (14).

The recording of the first 25 min started with the application of radionuclide, including image sequences of 45 × 1 s for perfusion, 97 × 15 s for secretion, and excretion. The injection of Furosemide 0.5 mg/kg of body weight maximum of 40 mg was given at time = 0.

The SRF as the parameter of the quality of the renal function was calculated. $\frac{1}{2}$Tmax was calculated for the assessment of ureteropelvicalyceal dilatation severity, $\frac{1}{2}$Tmax < 10 min was accepted as a cutoff point for normal kidneys, $\frac{1}{2}$Tmax between 10 and 15 min was considered moderate obstruction, and $\frac{1}{2}$Tmax > 15 min was significant obstruction. $\frac{1}{2}$Tmax was then compared with scintigraphy.

### Statistical Analysis

Descriptive analysis of categorical variables is presented as counts and percentages. A chi-square test (or Fisher exact when appropriate) was used to assess differences between the incidence of urinary tract anomalies in the US and MRU. The relationship between vpDRF vs. SRF and RTT vs. $\frac{1}{2}$Tmax was analyzed with a Bland and Altman plot and Spearman correlation. Statistical analysis was performed with SPSS version 25 (IBM, Armonk, NY) software. A *p*-value < 0.05 was considered significant.

## Results

The median age of the selected 25 patients (nine girls, 16 boys) with megaureters was 10 months (IQR: 3.35 years). No complications, such as anaphylactic or allergic reactions, were reported after MRI contrast medium. Thirty megaureters were found in 50 kidneys, mostly on one side, in the examined group of children. In five out of 25 children, megaureter was both-sided; in a single case, both poles of a duplex kidney were affected, however with incomplete duplication (ureteral bifidity). The sensitivity of US and MRU in diagnosing megaureter was comparable with 100% sensitivity of both methods. Megaureter was associated with duplex kidney in 16/30 kidneys (53%) and was also seen in 14 kidneys within a single collecting system (46%). Megaureter was associated with vesicoureteral reflux in seven cases. An ectopic ureter was found in six (24%) cases, three with duplex kidney and three with a single collecting system (Table 1).

**Table 1:** Summary of urological pathologies in the examined group.

	**Single collecting kidney**	**Duplex kidney**	**Total**
Megaureter	14 (28%)	16 (32%) (in one case both poles were involved)	30
Association with vesicoureteral reflux	8 (3 kidneys with refluxing megaureter)	4 (1 secondary to ureterocele incision)	12
Ureter ectopia	3	3	6
Kidneys after megaureter surgery	2 (in one case secondary megaureter after ureter reimplantation was diagnosed)	1 (after ureterocele incision)	3

**Figure 2:**
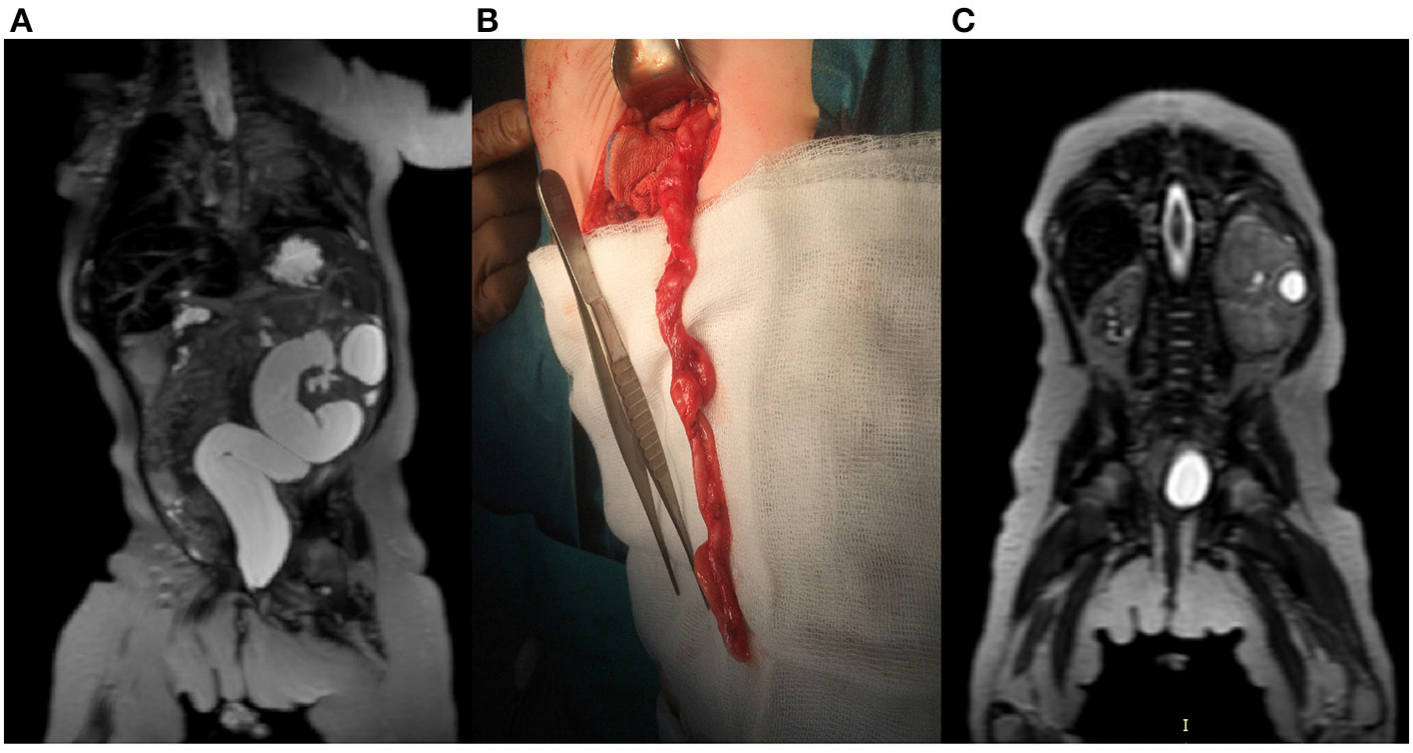
A 3-month-old female with left-sided megaureter. **(A)** T2-weighted image in the coronal plane shows left kidney with megaureter **(B)** Intraoperative picture of the partially excised megaureter. **(C)** T2-weighted image in coronal plane presents advanced dysplasia of the right kidney and localized dysplasia in the left, kidney.

We compared the sensitivity of the US and MRU in the diagnosis of the duplex kidney and ectopic ureter. Both methods achieved equal sensitivity in the diagnosis of ureterocele accompanying megaureter. The sensitivity of the US in evaluating duplex kidneys was 82% compared with 100% in MRU. Eighteen percent of false negative cases in US included duplex kidneys without dilatation of the collecting system. In the diagnosis of ureteral ectopia, MRU was significantly superior to US with sensitivity, respectively, 100 and 16%. The US technique showed limited value in the diagnosis of atonic/dysplastic ureteral segment as a cause of primary megaureter when compared with MRU. Four cases were visualized in MRU studies, whereas the US examination was negative for all of them (all confirmed during surgery) (Figures 2A,B). Renal scars and focal dysplasia were found in six kidneys in the analyzed group with 83% sensitivity for both MRU and US. One case was a false negative in each modality. Cystic lesions, suggesting focal dysplasia, were found in seven cases, and both methods showed sensitivity of 71% (Figure 2C).

Further detailed analysis of renal function based on ChopfMRU software and compared with scintigraphy reports (16 patients) are presented Table 2.

**Table 2:** Comparison of fMRU and dynamic MAG3 scintigraphy with classification the severity of obstruction basing scintigraphy.

**Parameter**	**Kidney**											
	**Normal**				**Moderate uropathy**				**Obstructive uropathy**			
	**(**$\frac{1}{2}$ **T max** ** <10 min)**				**(**$\frac{1}{2}$ **T max** **>10 min** ** <15 min)**				**(**$\frac{1}{2}$ **T max** **>15 min)**			
	***N***	**Range**	**Mean**	**Standard deviation**	***N***	**Range**	**Mean**	**Standard deviation**	***N***	**Range**	**Mean**	**Standard deviation**
RTT (min)	14	2.37–6.52	4.08	1.57	6	4.13–12.32	6.18	3.09	2	3.07–5.18	4.13	1.49
From Max to $\frac{1}{2}$ T max	12	2.81–7.19	4.78	1.74	6	8.16–11.90	9.71	1.39	2	14.20–21.40	17.80	5.09
vpDRF (%)	14	35.84–69.94	54.58	8.80	6	36.24–94.55	56.32	20.52	2	39.36–48.16	43.76	6.22
SRF (%)	14	38.50–66.20	52.31	7.23	6	33.80–98.40	55.57	22.08	2	39.40–46.10	42.75	4.74

*fMRU, functional magnetic resonance urography; SC scintigraphy RTT, renal transit time; Tmax, time to maximum enhancement; vpDRF, differential renal function based on volume and patlak number in fMRU analysis; SRF, split renal function; SD, standard deviation*.

**Figure 3:**
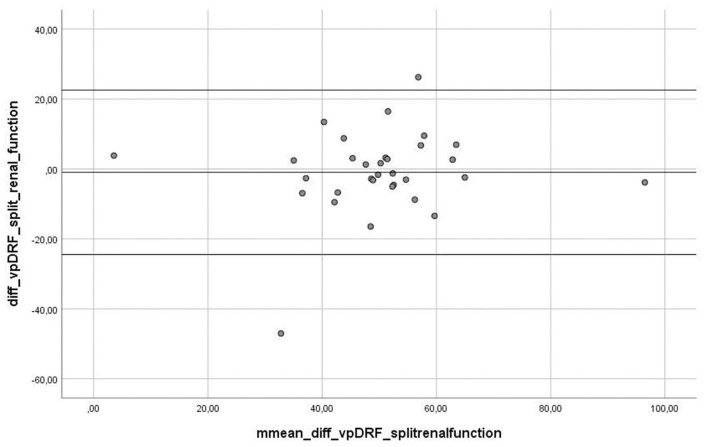
Graph presenting Bland-Altman plot of difference between measurement between two methods, fMRU and scintigraphy. The Linear Regression output showed no significant values, indicating no trend- both methods are changeable (*t* = 0.721, *p* = 0.477).

The range of value for vpDRF was 5.45–94.55% with the mean of 49.34%, whereas for scintigraphy, the range of SRF values was 1.63–98.40%, with the mean of 50.30%. For “vpDRF vs. split renal function”, no significant difference was detected between these methods (*p* = 0.657). For graphic analysis of comparability between both methods, we used Bland–Altman plots (Figure 3). The linear regression output showed no significant values, indicating no trend; both methods can be used interchangeably (*t* = 0.721, *p* = 0.477).

Severity of urinary tract obstruction was analyzed based on RTT and $\frac{1}{2}$ Tmax. In “RTT vs. T $\frac{1}{2}$ max” absolute values analysis, the two methods differed to a statistically significant level (*p* < 0.001) for a one-sample *t*-test for the difference, which clearly demonstrated these two measurements are significantly different from each other.

However, a Spearman correlation test showed moderate correlation between fMRU and scintigraphy in classifying as normal or moderate obstruction of the ureter (*r* = 0.441, *p* < 0.05). The third group with the severe obstruction was excluded from analysis due to the small number of affected kidneys. We noticed that, in nine kidney units, scintigraphy could not assess $\frac{1}{2}$ Tmax due to the presence overlaying radionuclide activity from the megaureter onto the region of the affected kidney, whereas fMRU was diagnostic in all them. Two of them were classified in fMRU as non-obstructive kidney, five were classified as moderate, and two as a severe obstruction.

## Discussion

The present study found comparable sensitivity of US and MRU techniques in the diagnosis of duplex kidney and megaureter. The sensitivity of US in the diagnosis of duplex kidneys was 84%; however, false negative results concerned kidneys without uropathy, unimportant clinically, whereas the sensitivity in the diagnosis of megaureter was equal between both methods. US exam seems to be a robust screening tool for these urinary tract anomalies, which is consistent with other recent reports (15–19).

In our study, the US technique showed limited value in the diagnosis of atonic/dysplastic ureteral segment as a cause of primary megaureter when compared with MRU as four cases, confirmed during surgery, were visualized in MRU alone. MRU enables evaluation of the morphology of megaureter stenosis, such as primary megaureter, ureteral ectopia, or ureterocele as well as assessment of the length of the ureter stenosis, and provides invaluable information for the surgical decision making, choosing an appropriate surgical approach, such as ureter reimplantation, laparoscopic or endourological treatment, such as endoscopic high-pressure balloon dilatation, endoureterotomy or temporary double-J stenting.

In our study, the most accented difference between both methods was found in the diagnostics of the ectopic ureter, indicating low sensitivity of US compared with MRU and not exceeding 16%. MRU seems to be a diagnostic tool of choice for ureteral ectopia, not only for the detection of this pathology, but also in the assessment of its severity and concomitant pathologies. The delayed diagnosis of the ectopic ureter, particularly when the obstruction is not significant, can cause recurrent urinary tract infections, bladder dysfunction, or prolonged urine incontinence, usually in females (20).

**Figure 4:**
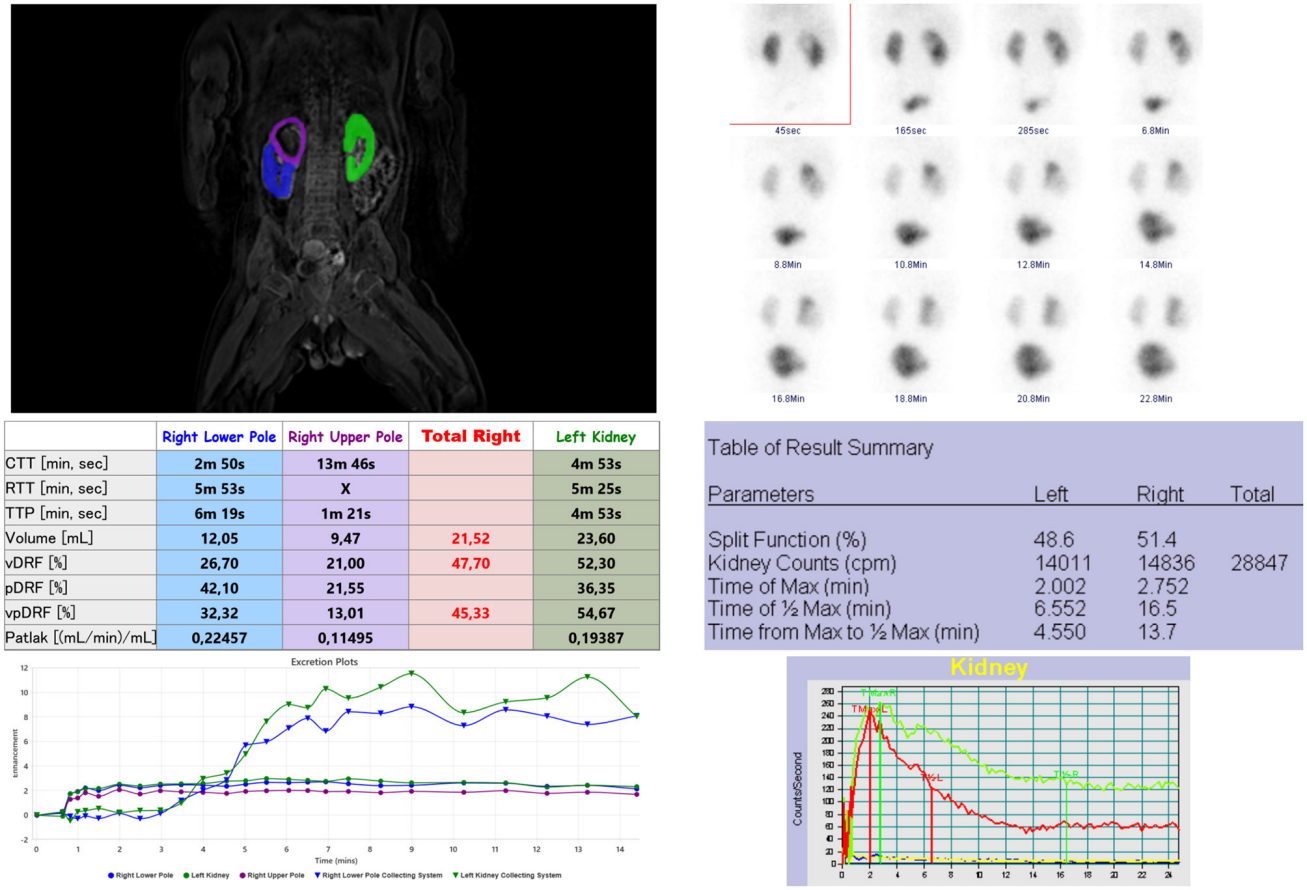
Analysis of renal functional MR urography (left) and scintigraphy (right) of a duplex right kidney with upper pole megaureter. There is graphic presentation of kidneys segmentations, absolute values of fMRU and scintigraphy parameters and at the bottom enhancement curves. In fMRU the enhancement curves and differential renal function (vpDRF) are taken separate for each moiety of right kidney. The image on the right presenting scintigraphy analysis - elongated time of T1/2Max of the right kidney due to radiotracer retention in upper pole.

We found a significant and strong correlation in the assessment of relative renal function by MRU (DRF) and scintigraphy (SRF) (*r* = 0.657, *p* < 0.001). In cases of duplex kidney, clinically relevant information of each collecting system can be obtained from MRU and differential renal function can be assessed for each moiety. In our study, we identified five such cases; in all of them, the differential renal function of each moiety was very important before the decision of heminephrectomy or corrective surgery (Figure 4). In many cases of megaureter and duplex kidney, the upper moiety is found dysplastic or hypofunctional, and it may be difficult to evaluate with precision in scintigraphy (17), which is limited in spatial resolution, whereas MRU allows observation of the flow of excreted contrast in the collecting system over time, even in the dilatated collecting system, with excellent resolution. MRU seems also to be an effective imaging tool in the visualization of complications caused by obstructive uropathy, including scarring or dysplasia of the parenchyma. In our study, we found scars or focal dysplasia in 10 kidneys with the use of MRU, the sensitivity of which was comparable to US.

fMRU presented moderate correlation in classification of the degree of ureteral obstruction compared with scintigraphy in normal and moderate obstructive kidneys. However, in nine kidney units, the parameter $\frac{1}{2}$ Tmax could not be calculated in scintigraphy due to radiotracer activity in tortious megaureter masking the renal parenchyma field and plana character of the method. Two of them were assessed in fMRU as non-obstructive and five as moderate obstruction in fMRU, which means that, in almost 40%, the scintigraphy results had limited diagnostic value. Further studies are necessary to optimize both methods; however, our results seem to be promising.

MRU as a urinary tract diagnostic tool is well accepted in the pediatric population; however, it is still limited to nephrology and urology centers in academic hospitals. MRU provides not only information about exact anatomy, but also renal function. MRU seems to be a unique tool as it provides information on kidney morphology (like US), the degree of kidney damage (like US and DMSA (dimercaptosuccinic acid) scintigraphy), but also on the function of kidney and its collection system (like MAG3 scintigraphy) (5, 6, 8, 9, 21–23). The basic limitations of fMRU are contrast agents that need to be used for functional analysis. Highly stable, macrocyclic gadolinium contrast agents are commonly used in children. They present with low incidence of side effects (0.04–3.8%), and severe side effects do not exceed 2/10,000, which is by far lower than for non-ionic iodinated contrast agents with adverse allergic reactions rate of 0.18% (24–26).

Sedation, necessary for fMRU in younger children is another limitation despite the fact that the most recent studies show limited negative influence of sedation shorter than 1 h (27, 28). The alternative, a promising approach called a feed-and-sleep technique of imaging in younger neonates, requires more time for preparation and is burdened with the risk of exam interruption or lower quality of images (29).

On the other hand, cumulative information obtained from the MRU can reduce the number of excretory urography and renal scintigraphy studies required, eliminating radiation exposure and speeding up the final diagnosis. Additionally, MRU can be helpful in the process of planning the surgery, especially in duplex kidneys and ectopic ureteral insertion (15, 18, 19). MRU gives an insight into the anatomy of the duplex kidney and allows precisely finding the plane of division between the poles to resect safely without fear of opening the phial-pelvic system of the remaining pole of the kidney. It also prevents leaving fragments of the resected pole, which prevents postoperative cysts from forming in the incision line. This is very important because more and more pediatric urological surgical interventions are performed using laparoscopic, less invasive techniques, and preoperative knowledge about morphology of the anomaly is crucial (15, 22).

The main limitations of the study are the relatively small numbers and retrospectively collected data.

In summary, our results underline the multidimensional role of MRU in the diagnostic algorithm of megaureters.

Based on our data, MRU is a universal method in the assessment of kidneys with megaureters, complementing the results of the US (urinary tract morphology) and enabling 3-D reconstruction, especially in children with suspicion of ureteral ectopic insertion. MRU offers a more accurate functional analysis of the renal function with higher spatial resolution analysis of the split renal function of affected parts of the kidney, especially in complex urinary tract anomalies. The advantages of MRU should update its role in the diagnostic algorithm of urinary tract anomalies, particularly in patients with megaureters, and may become a verifying method after sonographic screening.

## Data Availability Statement

The raw data supporting the conclusions of this article will be made available by the authors, without undue reservation.

## Ethics Statement

The studies involving human participants were reviewed and approved by Independent Bioethics Committee for Scientific Research at Medical University of Gdańsk. Written informed consent from the participants' legal guardian/next of kin was not required to participate in this study in accordance with the national legislation and the institutional requirements.

## Author Contributions

DŚ, MG, and PC contributed to conception and design of the study. DŚ, MG, and AD organized the database. MK performed the statistical analysis. DŚ wrote the first draft of the manuscript AG and MP wrote sections of the manuscript. All authors contributed to manuscript revision, read, and approved the submitted version.

## Conflict of Interest

The authors declare that the research was conducted in the absence of any commercial or financial relationships that could be construed as a potential conflict of interest.

## Publisher's Note

All claims expressed in this article are solely those of the authors and do not necessarily represent those of their affiliated organizations, or those of the publisher, the editors and the reviewers. Any product that may be evaluated in this article, or claim that may be made by its manufacturer, is not guaranteed or endorsed by the publisher.

## Abbreviations

MRI: magnetic resonance imaging
MRU: magnetic resonance urography
fMRU: functional magnetic resonance urography US &#8211
ultrasound
T2WI: T2 weighted images
RTT: renal transit time
CTT: caliceal transit time
DRF: differential renal function
pDRF: Patlak differential renal function
vDRF: volume differential renal function
SRF: split renal function
MAG3: ^99m^Tc-mercaptoacetyltriglycine.
